# Medical tourism and policy implications for health systems: a conceptual framework from a comparative study of Thailand, Singapore and Malaysia

**DOI:** 10.1186/1744-8603-7-12

**Published:** 2011-05-04

**Authors:** Nicola S Pocock, Kai Hong Phua

**Affiliations:** 1 Lee Kuan Yew School of Public Policy, National University of Singapore, 469C Bukit Timah Road, OTH Building, Singapore 259772, Singapore

## Abstract

Medical tourism is a growing phenomenon with policy implications for health systems, particularly of destination countries. Private actors and governments in Southeast Asia are promoting the medical tourist industry, but the potential impact on health systems, particularly in terms of equity in access and availability for local consumers, is unclear. This article presents a conceptual framework that outlines the policy implications of medical tourism's growth for health systems, drawing on the cases of Thailand, Singapore and Malaysia, three regional hubs for medical tourism, via an extensive review of academic and grey literature. Variables for further analysis of the potential impact of medical tourism on health systems are also identified. The framework can provide a basis for empirical, in country studies weighing the benefits and disadvantages of medical tourism for health systems. The policy implications described are of particular relevance for policymakers and industry practitioners in other Southeast Asian countries with similar health systems where governments have expressed interest in facilitating the growth of the medical tourist industry. This article calls for a universal definition of medical tourism and medical tourists to be enunciated, as well as concerted data collection efforts, to be undertaken prior to any meaningful empirical analysis of medical tourism's impact on health systems.

## Introduction

Growing demand for health services is a global phenomenon, linked to economic development that generates rising incomes and education. Demographic change, especially population ageing and older people's requirements for more medical services, coupled with epidemiological change, i.e. rising incidence of chronic conditions, also fuel demand for more and better health services. Waiting times and/or the increasing cost of health services at home, coupled with the availability of cheaper alternatives in developing countries, has lead new healthcare consumers, or medical tourists, to seek treatment overseas [1]. The correspondent growth in the global health service sector reflects this demand. The globalisation of healthcare is marked by increasing international trade in health products and services, strikingly via cross border patient flows.

In Southeast Asia, the health sector is expanding rapidly, attributable to rapid growth of the private sector and notably, medical tourism, which is emerging as a lucrative business opportunity. Countries here are capitalising on their popularity as tourist destinations by combining high quality medical services at competitive prices with tourist packages. Some countries are establishing comparative advantages in service provision based on their health system's organizational structure (table 1). Thailand has established a niche for cosmetic surgery and sex change operations, whilst Singapore is attracting patients at the high end of the market for advanced treatments like cardiovascular, neurological surgery and stem cell therapy [2]. In Singapore, Malaysia and Thailand alone, an estimated 2 million medical travellers visited in 2006 - 7, earning these countries over US$ 3 billion in treatment costs (table 2).

**Table 1:** Health systems in comparison [3]

Country	Thailand	Malaysia	Singapore
Organizational structure	Pockets of excellence in some private Bangkok hospitals	Growing private health sector with movement of qualified workforce	Balanced public-private mix, corporatized public sector
			
National strategy	Regional health hub	Industrial strategy to develop tourism	Economic growth strategy to develop biomedical industries
	Extensive tourism infrastructure		Regional service hub
			Medical R&D support
			
Policy impact	Issues of growing inequity and urban-rural divide	Public-private divide	Narrow income gaps of public and private sectors
		Racial inequities between public and private sectors	

**Table 2:** Export of health services [2,4,5]

	Estimated earnings	No. foreign patients	Origin of patients (in order of volume)	Specialty
**Thailand (2006)**	Baht 36 billion (US$ 1.1 billion)	1.4 million	Japan, USA, South Asia, UK, Middle East, ASEAN countries	Cosmetic and sex change surgery
**Singapore (2007)**	S$ 1.7 billion (US$ 1.2 billion)	571 000	Indonesia, Malaysia, Middle East	Cardiac and neuro surgery, joint replacements, liver transplants
**Malaysia (2007)**	253.84 million MYR (US$78 million)	341 288	Indonesia, Singapore, Japan, India, Europe	Cardiac and cosmetic surgery

Carrera and Bridges (2006) define medical tourism as "the organized travel outside one's natural healthcare jurisdiction for the enhancement or restoration of the individual's health through medical intervention", using but not limited to invasive technology. The authors define medical tourism as a subset of health tourism, whose broader definition involves "the organized travel outside one's local environment for the maintenance, enhancement or restoration of the individual's wellbeing in mind and body". Importantly, their definition of medical tourism takes into account the territorially bounded nature of health systems, where access to healthcare is often but not always limited to national boundaries [6]. Medical tourism constitutes an individual solution to what is traditionally considered a public (government) concern, health for its citizens, who at the micro level are responding to market incentives by seeking lower cost and/or high quality care overseas that cannot be found at home. These tourists may be uninsured or underinsured. Travelling overseas for medical care has historical roots, previously limited to elites from developing countries to developed ones, when health care was inadequate or unavailable at home. Now however, the direction of medical travel is changing towards developing countries [7], and globalization and increasing acceptance of health services as a market commodity [8] have lead to a new trend; organized medical tourism for fee paying patients, regardless of citizenship, who shop for health services overseas using new information sources, new agents to connect them to providers, and inexpensive air travel to reach destination medical [9].The impact of medical tourism on health systems is as yet unknown due to a dearth of data and empirical analysis of the phenomenon.

Governments are noticeably playing a strong marketing and promotional role in the emerging medical tourism industry. This is a clear trend in Southeast Asia, especially in Thailand, Singapore and Malaysia, the main regional hubs for medical tourism, where medical tourist visas are available and government agencies have been established with the mandate to increase medical tourist inflows [10]. Governments in Indonesia, the Philippines and Vietnam have also expressed interest in promoting the industry. The potential economic benefits of medical tourism make it an attractive option for governments. Medical tourism can contribute to wider economic development, which is strongly correlated with improved population health status as a whole, e.g. increased life expectancy, reduced child mortality rates [11]. Encouraging foreign direct investment in healthcare infrastructure and medical tourist inflows with correspondent revenue can create additional resources for investment in health care [12]. Furthermore, medical tourism may slow or reverse the outmigration of health workers, particularly of specialists [13].

However, health systems in some of these countries face challenges in ensuring basic health service coverage for their own citizens [3]. Two tier healthcare provision has emerged in Malaysia, with private services limited to those who can afford it and public services for the rest of the population [14]. Thailand's public to private health worker brain drain has strained public health provision, especially in rural areas [15,16]. Trade in medical supplies, organs, pharmaceuticals and health worker migration have dominated policy debates about the impact on health systems in developing countries, including concerns about intellectual property rights and access to affordable drugs, the latest medical technology, and retaining doctors and nurses within the public sector and/or within the country's health system at all. There are growing concerns about the impact of medical tourism on health systems, particularly equity of access for both foreign and local consumers [17]. Inequities at home, either by low quality services and/or inability to pay, prompt people to seek cheaper and high quality care treatment overseas. As Blouin (2010) contends, a policy question that remains unanswered is whether medical tourism can improve the capacity of poor people in developing countries to access health services. She calls for the exploration of policy mechanisms that mitigate the risks associated with medical tourism, whilst harnessing the potential benefits, for local consumers [18].

In the academic literature, conceptual analyses of medical tourism have emerged from a tourism management perspective, analysing supply and demand factors [19-22], and as a node in the trade in health perspective [10,23-26]. Legal literature is beginning to cover patient liability issues when surgery is carried out overseas [27]. Recent work has begun to analyse medical tourism and its potential impact on health systems in specific countries [1,28,29]. Yet not all health systems functions are analysed in these accounts. A core concern is whether medical tourism diverts resources from public components of health systems in destination countries [30]. Furthermore, conceptual frameworks in the health systems literature focus on the impact of targeted, vertical interventions in health systems [31]. But medical tourism is a phenomenon rather than an intervention; its policy implications have yet to be considered within the context of a health system.

This paper presents a conceptual framework of medical tourism and policy implications for health systems in Southeast Asia, drawing on the cases of Thailand, Singapore and Malaysia, via an extensive review of the academic and grey literature, as well as insights from health consultancies in the public and private sectors across the region. This framework provides a basis for more detailed country specific studies on the benefits and disadvantages of medical tourism, of special relevance for policymakers and industry practitioners in other Southeast Asian countries with similar health systems where governments have expressed interest in facilitating the growth of the medical tourist industry. Bridging the social science disciplines, the public policy approach to research is a pragmatic one, with the end goal of translating research into useful policy recommendations, in this instance those that optimise the benefits of medical tourism for both foreign and local consumers and mitigate the risks. Research methodology is outlined below, followed by the policy implications of medical tourism for health systems at their governance, delivery, financing, human resources and regulation functions [32,33]. The conclusion emphasizes the need for concerted data collection efforts and identifies variables for further analysis of medical tourism's potential impact on health systems.

## Research methodology

Media reports on the medical tourism industry and participation in regional conferences enabled the researchers to pinpoint Singapore, Thailand and Malaysia as the three main hubs for medical tourism in Southeast Asia for comparative analysis. Broadly, there are four types of comparative health policy analyses. The first constitute descriptive studies, with no hypothesis or testing of explanations on why patterns exist, leaving policy explanations implicit for the reader to gauge. The second include collections of international case studies with some assessment of performance, whilst the third type includes studies employing a common framework for analysis (e.g. privatization). The fourth type of cross national studies are those that show a fundamental theoretical orientation, with a specific theme or question as a focus of analysis (Marmor et al 2005: 341 - 2) [34]. We decided to undertake this fourth type of comparative analysis, in order to generate a conceptual framework that could be usefully employed by policymakers to understand the policy implications of medical tourism on health systems with similar structures. Methods employed focussed on conceptualising rather than describing, where one or more new concepts are developed to explain what is being studied [35]. An inductive, theory building approach [36] is appropriate to examine medical tourism where knowledge is far lacking, especially in relation to health systems.

An initial informal literature scan using the search criteria "medical tourism AND Asia" in google scholar revealed a lack of data and authoritative sources on medical tourism, particularly figures for number of patients and estimated earnings. Academic literature was searched exhaustively in the PubMed and Social Science Research Network databases using the search criteria "medical tourism AND Asia" (92) and "medical travel AND Asia" (806), generating a range of mostly conceptual research. Abstracts were scanned for reference to Thailand, Singapore and Malaysia and/or reference to health systems in general. Additional articles were located using the reference list of selected articles. Study selection was not systematic; no article was omitted but considered in the context of health systems/medical tourism in Asia (43). Articles gathered were then categorised according to content focus (e.g. privatisation of health systems, medical tourism empirical evidence, health and trade nexus). Following categorisation, all articles were analysed to identify medical tourism interaction points across the health system functions, with new material continually brought into the analysis. Concurrent to the theory building process, quantitative data on the nature of health systems in the three study countries were retrieved from official country sources and the World Health Organization. These data were triangulated with the academic literature to validate claims made about the nature of health systems. This data also enabled the researchers to make systematic comparisons between the three country health systems. Following this step, grey literature were searched using the above search criteria in Factiva, a news item database, to provide examples of recent developments in the medical tourist industry in the three study countries. Other grey literature sources included management consultancy research reports, working papers on medical tourism, and medical tourism industry player's statistics and promotional materials. Subsequent to analysis and identification of the conceptual framework, potential policy options were outlined based on the literature and/or innovative examples of comparative health policy responses in the region. We anticipated that the different nature of health systems (e.g. mostly public versus private delivery) would also generate differential policy implications according to local context. In the course of our comparative analysis, we found this to be the case to a large extent; however, medical tourism poses potential risks and benefits regardless of the current nature of a health system. As a phenomenon, it can fundamentally change the nature of health systems themselves without policy intervention (e.g. shift towards a dominantly private hospital sector). Thus, the policy implications described are broadly applicable to health systems in general, but of particular relevance to policymakers and industry practitioners in other Southeast Asian countries where governments have expressed an interest in developing the medical tourist industry.

## Results

### Governance in separate domains of trade and health

Medical tourism straddles the policy domains of trade and health. Its rise is situated within the rapid growth of trade in health services, driven by increased international mobility of service providers and patients, advances in information technologies and communications, and an expanding private health sector [10]. Trade by definition is international, but health systems (financing, delivery and regulation) remain nationally bounded. Additionally, trade objectives of increased liberalisation, less government intervention and economic growth generally do not emphasize equity, whereas health sector objectives like universal coverage do. Consequently, actors in the trade and health policy spheres tend to have conflicting objectives, and trade and health governance processes remain relatively separate at three levels; the international (World Trade Organisation (WTO) and World Health Organisation (WHO)), regional (Association of South East Asian Nations (ASEAN)) and national (government ministries). Reconciling the aims of economic growth with equitable health service provision and access makes governance of medical tourism within a country's health system challenging at best and contradictory at worst.

At the international level, there are clear tensions between the goals of protecting and promoting health and generating wealth through trade [23]. Trade and health policy negotiations occur in isolation, despite the growing importance of the trade and health nexus at the global level, e.g. extensive health worker migration and cross border consumption of health services (medical tourism) [10,23]. WTO membership requires adherence to a multitude of legally binding obligations, including removal of tariff and non tariffs barriers on goods and services. The WTO's formal governance architecture is embodied in its legally binding trade agreements and compulsory legal dispute mechanism. These legal apparatus afford it more compliance clout than the WHO, which by contrast is an advocacy organization. The WHO imposes no legal obligations on members, relies on non binding agreements, and has no compulsory dispute mechanism. Thus enforcement capacity in cases of non compliance to WHO agreements is limited [23]. Economic growth and trade considerations are likely to surpass health objectives at the global level when countries face sanctions or legally punitive measures for non compliance with trade agreements. Examples of trade and health policy incoherence include patents on essential medicines and tobacco promotion in developing countries, permitted by trade agreements [37].

Whilst most trade in health services takes place outside the framework of existing trade agreements, whether bilateral or multilateral [25], trade in health services including medical tourism is officially provisioned for under the General Agreement on Trade in Services (GATS). The four modes of supply include; 1. The cross border supply of services (remote service provision, e.g. telemedicine, diagnostics, medical transcriptions), 2. Consumption of services abroad (medical tourism, medical and nursing education for overseas students) 3. Foreign direct investment (e.g. foreign ownership of health facilities) and 4. Movement of health professionals [7]. Countries can choose to make GATs commitments (which legally bind them to open markets under the auspices and protection of the WTO) sectorally or via a specific mode. In ASEAN, only Cambodia, Malaysia and Vietnam have made GATs commitments relevant to the health sector [38]. Medical tourism is becoming bureaucratized, formalized and normalized [17] evidenced by GATs provisions for the health sector. In the context of increasing cross border trade in health services, governments have the option to either schedule GATs commitments in health or continue to trade outside of formal agreements. With rapidly changing domestic and international health markets, the latter looks likely, but it is worth noting that GATS commitments can also limit the degree to which foreign providers can operate in the market [39]. In policy terms, this clause can protect health systems from monopolization by foreign investors in the health sector.

Regionally, trade also tends to trump health in terms of policy action. ASEAN is primarily a trade forum, and the 1995 ASEAN Framework on Agreement on Trade in Services (AFAS) makes provisions for services liberalisation between members beyond the WTO GATs. Unlike the WTO, ASEAN has no legal authority to enforce compliance, but a dispute settlement mechanism was recently signed. Whilst the health sector is not covered under the AFAS, it is envisioned that the free flow of all goods, services, investments, capital and skilled labour will be achieved to create an ASEAN Economic Community (AEC) by 2020 [40,41]. The ASEAN Economic Community (AEC) council meets bi annually to work towards deepening and broadening regional economic integration. In contrast, the ASEAN Health Minister's Meeting (AHMM) is held every two years. Currently, ASEAN health cooperation is limited to disaster preparedness for natural disasters and infectious disease outbreaks. Agreements in health are limited to sanitary and phytosanitary measures, bar a non legally binding Mutual Recognition Agreement (MRA) on the movement of health professionals. The ASEAN Work Plan on Health Development (2010 - 2015) was finalised in July 2010 to cover broader regional health issues, including non communicable diseases, maternal and child health and primary health care [42,43]. Despite ASEAN's regional economic and health integration, there have been no agreements signed concerning the medical tourism industry. Foreign direct investment by regional players in neighbouring countries is accelerating, with private companies like Singapore's Parkway Holdings (one of the largest hospital operators in Asia) and the Raffles medical group acquiring hospitals in Singapore, Malaysia, Brunei, India and China [26]. Malaysia's state investment company Khazanah's $2.6 billion bid in Parkway Holdings in 2010 gave it a 95% stake in the company [44]. Foreign investment by both private and state investment companies implies that significant profits can be made in the health sector of other countries, with profits accruing to shareholders overseas and few benefits for local consumers, unless profits are taxed and reinvested in the destination health system. The substantive economic capacity of these regional players means that health policy aims, like universal access to healthcare, are likely to come secondary to trade policy aims, like increasing foreign investment that can be gained from medical tourism.

Trade and health policy incoherence in promoting both medical tourism and universal coverage for local consumers at the national level is evident. Whilst several studies on medical tourism allude to government's role in promoting medical tourism [8,16,21], these do not differentiate between the role of *different *government ministries and their respective policy aims. Trade and tourism ministries are primarily concerned with increasing economic growth and facilitating international trade in the services sector. In contrast, a health ministry's aim is to improve overall population health and ensure equity in health service access and delivery. Health systems are also nationally bounded; maximising scarce public resources for health within given territorial constraints gives rise to healthcare protectionism by governments, typified by strict eligibility requirements for access to state subsidised services by migrants. Whilst expansionist medical tourism policies had been initiated in trade and tourism ministries of all three countries, there appears to be a spill over effect on ministries of health (MOH). Increasingly, MOH's are establishing medical tourism committees and departments, dedicated to the promotion of their respective countries' health facilities to other governments/foreign patients. For example, Thailand's medical hub policy was initiated in 2003 by the government agency the Thailand Board of Investment, whilst the Ministries of Commerce, Department of Export Promotion and the MOH in collaboration with private hospitals are now the main implementers of the policy [15]. Whilst Malaysia's national health plan does not mention medical tourism as a strategic aim [45], the MOH formed an inter-ministerial committee for the promotion of medical and health tourism (MNCPHT) in 2003 [28]. Of the three countries, Singapore's government agencies have the most integrated policy stances that strongly support medical tourism [2], reflective of the country's prioritisation of economic growth. Singapore's Tourism Board, the Ministry of Trade and Industry's Economic Development Board and the MOH have set a target to attract 1 million foreign patients by 2012 [46], whilst one of the MOH's explicit priorities is to "exploit the (country's) economic value as a regional medical hub" [47]. In 2004, a multiagency government initiative (including the MOH) SingaporeMedicine was launched with the aim of developing Singapore as a medical hub. Whilst trade and tourism and health ministry objectives are not easily reconciled, medical tourism growth provides an opportunity for inter ministry policy coordination, e.g. via a cross subsidization mechanism whereby medical tourist revenues are taxed, providing extra income for public hospitals. In the three countries, an apparent convergence in trade, tourism and health ministry priorities is taking place, reflective of growing acceptance of health as a private good globally. Improved data collection on medical tourist flows and health systems use and access by local consumers are necessary to assess whether policies that promote medical tourism and universal coverage are reconcilable. Pre-emptively, government ministries should work towards more integrated governance of medical tourism, especially given the highly privatised health system landscape and existing inequities in health systems use and access by local consumers, which could be aggravated by foreign patient inflows.

### Delivery in private versus public sector

Medical tourism is driven by the for profit private sector in health systems. The private sector dominates primary care provision in Singapore and Malaysia, but is slowly expanding its role in tertiary hospital care. Private primary care providers are concentrated in urban areas, with public primary care providers catering to those in rural areas, as seen in Thailand and Malaysia [14,48]. Hospital services are dominated by the public sector, with a 70 - 80% share of beds (table 3) but private hospital providers are steadily growing. In Thailand, private hospital numbers have hovered consistently at 30% of total hospitals between 1994 and 2006 [48]. In Singapore, private sector hospital growth has risen in proportion with public sector hospital growth between 1998 and 2008 [49]. Private hospitals are smaller in size and tend to be located in urban areas, serving middle to high income patients as well as foreign patients [50]. In general, the public private mix of healthcare provision in this region reflects the country's level of economic development. During economic growth periods, wealthier populations have emerged with demand for private providers in response to perceived lower quality public provision. Consequently the public sector has become more pro poor as this group cannot afford private care, leading to the development of a two tier healthcare system seen in Thailand and Malaysia [14,51]. Public services are generally perceived to be of low quality or unresponsive in this region by local consumers [5052]. The steady growth of private sector hospitals has mirrored the increase in medical tourism (tables 2 and 3).

**Table 3:** Public versus private health provision [49,53-55]

	Hospitals		Beds		Beds per 1000 population	Primary care clinics	
		
	Public (%)	Private (%)	Public (%)	Private (%)		Public	Private
**Thailand**	67.9% (2007)	32.1% (2006)	69.3% (2006)	30.7% (2006)	2.2 (2002)	80.5% (2007)	19.5% (2006)
**Singapore**	63.6% (2009)	36.4% (2009)	80.6% (2009)	19.4% (2009)	3.2 (2007)	1.5% (2005)	98.5% (2005)
**Malaysia**	40.6% (2008)	59.4% (2008)	77.9% (2008)	22.1% (2008)	1.8 (2007)	32.1% (2008)	67.9% (2008)

The link between a growing private, for profit sector that caters to medical tourists and access to such services by local consumers without the ability to pay is elusive. Private ownership of health facilities means that benefits accrued (profits from service fees for foreign patients) are remitted offshore to companies based in different countries who are investing in private hospital chains across Southeast Asia. For example, the recent Fortis-Parkway merger of the second largest Indian healthcare group with the largest private Singapore-Malaysia group created the largest hospital chain in Asia. Parkway's subsequent take-over bid by Malaysia's state investment company Khazanah, means that profits accrued are remitted to Malaysia for health services rendered in Singapore and India. Purchase of costly technology that doesn't have a wider social benefit for the procedures that medical tourists demand has raised concerns about "crowding out" local consumption of high technology procedures [12]. Furthermore, government subsidies for private sector growth, via tax breaks and preferential access to land, is unlikely to benefit the health system at large nor facilitate broader public health goals (universal coverage) if private hospitals cater to larger shares of fee paying, foreign patients. This can be seen in Malaysia, where tax incentives are available for building hospitals (industry building allowance), using medical equipment, staff training and service promotion (deductions on expenses incurred) [8]. Private sector growth in health is implicitly encouraged via these benefits, at the same time as government construction of new hospitals has stalled due to alleged insufficient public funds [56].

Medical tourism is emerging in public sector hospitals at the same time as it is being driven by the private sector, notably in corporatized (public) hospitals. Corporatization of hospitals in Singapore since 1985 granted hospitals greater autonomy and exposure to market competition under government ownership, with the aim of lowering costs and improving service quality [57]. All public hospitals in Singapore are Joint Commission International (JCI) accredited [58]. Given that these hospitals are publicly owned, revenues accruing to medical tourism are taxable and thus profits can be reinvested back into the public health system by the government. In Malaysia and Thailand, some public hospitals are allowing their surgeons to operate a private wing for private patients, including medical tourists. This policy move could incentivise surgeons to treat the additional fee paying foreign patients over local consumers, when public health resources are already strained in those countries.

The majority of medical tourists in Southeast Asia hail from neighbouring countries, reflecting inequities in service provision at home, either via unavailability of quality services or underinsurance. In Singapore and Malaysia, most medical tourists are from ASEAN countries, whilst Thailand's consumers are often from outside the region, with the Japanese accounting for the largest share of foreign patients (table 2) [50]. Indonesians travel to Singapore and Malaysia for medical treatment, whilst Cambodians cross the border to Vietnam for higher quality health services. Low quality public and private health provision at home forces them to leave for overseas treatment. Cost is a factor, but Malaysian, Singaporean and Thai hospitals offer specialised services unavailable in other, especially poorer, ASEAN countries [2,50]. The policy implications go beyond the potential to crowd out consumption by locals. As Chee (2010) points out, when middle class fee paying patients decide to undertake treatment abroad, their domestic health systems lose out, not only financially but in terms of the political pressure that these potential consumers could exert to improve the health system that poorer consumers rely upon [28]. The possibility to "exit" low quality health systems gives the middle class little incentive to exert pressure for quality improvement [59]. Policy options that raise quality standards and minimize quality differentials, both within and between countries in Southeast Asia, would benefit both foreign and local consumers. These include public private linkages via professional exchanges, joint training initiatives, shared use of facilities between public and private providers to maximise resource use, telemedicine, and use of complementary/specialised treatments [1,12].

### Healthcare financing and consumerism

Consumer driven healthcare is becoming the normalised globally and in this region, partly encouraged by governments and the private sector seeking to shift responsibility for one's health to the individual in response to rising healthcare costs and demand for services. Singapore and Malaysia exemplify this trend, as public health expenditure has slowly been declining whilst private health expenditure has increased [28]. The Thai government spent almost double the amount on health as a percentage of total government expenditure (14.1%) compared to Singapore (8.2%) and Malaysia (6.9%) in 2008 [53]. As table 4 shows, the Thai government contributes the majority of total health spending (75.1%), in contrast to Malaysia and Singapore, where private health spending surpasses government health spending. Although both Singapore and Malaysia in theory offer 100% population coverage, high out of pocket payments (OPPs) suggest effective coverage is less than this [52]. Both countries are encouraging greater use of individual financing instruments to pay providers, in addition to compulsory state insurance schemes (Medishield in Singapore) or taxation (Malaysia). These include medical savings accounts (Medisave in Singapore, Employee Provident Fund Account 2 in Malaysia) [60] and widespread private insurance. Thailand is the exception, where the government's commitment to enrolling the population in its universal social insurance scheme means that government investment in health has risen since 2002 [56,61,62].

**Table 4:** Health expenditure [53]

	Total health expenditure as % of Gross Domestic Product (2008)	Government expenditure on health as % of total government expenditure (2008)	Government health expenditure as % of total health expenditure (2008)	Private expenditure as a % of total health expenditure (2008)	Out of pocket expenditure as a % of private health expenditure (2008)	Private prepaid plans as a % of private health expenditure (2008)
**Thailand**	4.0%	14.1%	75.1%	24.9%	71.1%	20.9%
**Singapore**	3.4%	8.2%	35.0%	65.0%	93.9%	2.8%
**Malaysia**	4.3%	6.9%	44.1%	55.9%	73.2%	14.4%

The most regressive financing mechanism, out of pocket payments (OPPs), dominates private health spending in all three countries. More OPPs for services leads to more competition in private healthcare markets, as providers are more likely to compete for patients based on price, especially given the price transparency made possible by the internet. Medical tourist payments are dominated by OPPs, but these payments are becoming more organized as part of insurance coverage. For example, since March 2010 Singapore's Medisave can be used for elective hospitalizations and day surgeries in hospitals of two partner providers in Malaysia, Health Management International and Parkway Holdings [63]. Deloitte's 2009 medical tourism industry report highlighted four US health insurers who are piloting health plans that permit reimbursement of elective procedure overseas in Thailand, India and Mexico [64]. The trend of insurance companies and employers turning to foreign medical providers to reduce costs looks set to continue as the medical tourism industry grows [29].

One policy implication of the increase in medical tourists on health financing is that differential pricing for foreign patients could drive up costs of services for local consumers over time. Redistributive financing mechanisms may offset these increases. Policy options include taxing medical tourist revenues to be reinvested in the public health system [12], expanding financing instruments that do not tie access to ability to pay (taxation, social insurance) and mandating private providers to participate in schemes that provide coverage to local consumers. Private hospitals could provide services to a specified percentage of foreign patients and local consumers enrolled in state schemes, or provide certain specialist treatment for locals (depending on a centre's area of clinical expertise). The need for such policies is pressing when, for example, private hospitals treating foreign patients in Thailand currently do not participate in social health insurance schemes, which covered 98% of the population in 2009 [25,52,65].

### Human resources and specialists

Health worker shortages persist to varying degrees in Southeast Asia, at the same time as demand for health services from foreign patients is rising. Whilst all three countries have health worker densities above the WHO critical threshold of 2.28 health workers per 1000 population, all countries face pressures to supply trained health workers to meet population health needs [66,67]. There are low doctor-to-patient ratios in Thailand and Malaysia (table 5), as well as continual outmigration of doctors from Singapore and Malaysia. Within ASEAN, these two countries record the highest levels of doctor outmigration to OECD countries [68]. International outmigration from Thailand is low, but intra-country migration from rural to urban areas and maldistribution of health workers is common [15,16]. In response to shortages, Singapore has been able to attract health workers from the Philippines and Malaysia. In Thailand, health workers must pass medical exams in Thai, limiting potential for physician immigration to the country. Whilst the foreign medical workforce inflow to Malaysia has been substantial, this has been insufficient to offset the outflow of Malaysian doctors to other countries [25].

**Table 5:** Human resources for health [49,53,69]

	Doctors per 1000 population	Doctors		Nurses per 1000 population	Nurses	
				
		Public (%)	Private (%)		Public (%)	Private (%)
**Thailand**	0.4 (2000)	78.4% (2005)	21.6% (2005)	2.8 (2000)	87.8% (2005)	12.2% (2005)
**Singapore**	1.5 (2003)	54.8% (2009)	45.2% (2009)	4.5 (2003)	68.5% (2009)	31.5% (2009)
**Malaysia**	0.7 (2002)	60.1% (2008)	39.9% (2008)	1.8 (2002)	71.2% (2008)	28.8% (2008)

Rising demand for health services in the region has precipitated the growth in private medical and nursing schools across Southeast Asia and correspondent rise in trained health workers. Public and private medical schools in the region are establishing partnerships with reputable universities overseas. Thailand's Mahidol university nursing department has established links with nursing schools in Sweden, Canada, Australia, Korea, the UK and the USA to facilitate student and teaching exchanges. Singapore's National University recently opened a graduate medical school with Duke university in the USA, and Malaysia's Sunway university medical school trains students in partnership with Monash university in Australia. Such partnerships facilitate capacity building in human resources for health, as well as access to new markets for universities overseas. Importantly, these partnerships signal *quality *of human resources, crucial to the promotion of medical tourism [17].

Developing the medical tourism industry can be seen as a tactic to reduce international emigration of health workers, particularly of specialists. Anecdotal evidence from Thailand indicates that medical graduates, having acquired specialised medical degrees abroad, are finding it lucrative and more satisfying to stay in their home country [2]. Politicians in Singapore have reasoned that in order to recruit and retain specialists in a country with a small local population, that the country must attract a high volume of medical tourists. However, within countries, the growth of medical tourism may exacerbate public to private sector brain drain, notably of specialists who provide elective surgeries demanded by foreign patients. Whilst the proportion of doctors working in the public sector is higher than in the private sector in medical tourist countries (table 5), dual practice, whereby doctors combine salaried, public sector clinical work with fee for service private clientele [70], is common amongst specialists in Thailand and Malaysia. Retaining public sector specialists has become a challenge with the prospect of higher salaries and lower workloads in the private sector. Singapore has managed to maintain competitive public sector salaries, but in Thailand and Malaysia, with larger public - private pay discrepancies, medical tourism has the potential to further incentivise specialists to shift to the private sector. Evidence from Thailand suggests that medical tourism is not negatively impacting the health system by pulling doctors from rural areas. Rather, specialists from teaching hospitals in urban areas are shifting to private hospitals catering to foreign patients [67,71]. All three countries have a high number of doctors with specialty training e.g. 77.5% in Thailand in 2006, [48]. But these specialists are concentrated in the private sector; in Malaysia, only 25 - 30% of specialists work in the public sector [72]. Singapore is the exception, where 65% of specialists are in the public sector [73]. The type of surgery matters; for local consumers seeking specialist, essential surgery (e.g. cardiac, transplantation procedures), paying to see a specialist in a private hospital may be the only option. High quality, specialised care is typically provided in private hospitals and can only be afforded by middle to high income patients [50].

Medical tourism could exacerbate already endemic public to private brain drain in the region. A related concern in Thailand is that medical education is largely publicly funded; private hospitals do not share the costs of such education, yet hire from the same pool of graduates as the public sector [50]. Policy options to mitigate internal brain drain include instituting capitation payments for health costs and standard fees for doctors, regardless of whether a patient is local or foreign. Offering higher salaries in the public sector and bonding publicly funded graduates are options for governments (all three countries bond their graduates for between 3 to 5 years). Dual practice of specialists could be allowed but regulated, so that specialists dedicate a specified amount of time to treat local consumers. When public funds are used to train specialists who then shift to the private sector (potentially to treat medical tourists), redistributive government regulations like paying a fee to leave the public sector (Thailand) may plug a short term financial resource gap, but recruitment and retention is a persistent problem in this region.

### Regulation of quality control and new actors

Private hospitals in the three countries are accredited via different channels, leading to differing quality standards between public and private hospitals. Private hospital associations encourage industry self regulation, whereas public hospitals are regulated by the MOH or quasi governmental bodies. For example, publicly owned corporatized hospitals in Singapore operate with autonomy in a competitive environment, but government ownership allows them to shape hospital behaviour without cumbersome regulation [74].

Joint Commission International (JCI) is the most established medical tourist industry accreditor worldwide. Of the three profiled countries, Singapore has the highest number of JCI accredited providers (18), followed by Thailand (13) and Malaysia (7) [58]. JCI accreditation is an important quality signal to attract medical tourists, but this process is voluntary. The differing quality accreditation channels at the national (private hospital associations vs. MOH) and international levels may lead to inequitable quality standards between the public and private sectors, whereby private hospital standards surpass those in public hospitals, reflective of the current situation in low to middle income countries in Southeast Asia. This has implications for the quality of care received by local consumers without the ability to pay for private services, and the potential divergence of health outcomes between private fee paying patients (foreign and local) and those that can't afford such services. Malaysia's Society for Quality in Health (MSQH), a joint regulatory body launched by the Ministry of Health, Association of Private Hospitals of Malaysia and the Malaysian Medical Association, was recently awarded international accreditation by the ISQua on par with JCI. As the MSQH covers both public and private hospitals, this kind of international standard setting for both sectors could provide a regulatory template for other countries pursuing medical tourism, in order to ensure that both local and foreign consumers enjoy similar quality standards. Policy options include common standards for public and private providers [1] regulated by government, as well as compulsory JCI accreditation for hospitals catering to medical tourists.

New brokers that arise between hospitals and patients are proliferating rapidly. These agencies are located in developed and developing countries, connecting prospective patients to providers via the internet. As yet, the medical brokerage industry has no codes of conduct, and the lack of medical training of brokers raises questions about how these new actors evaluate quality of care when choosing which facilities to promote to prospective patients. There are also no explicit formal standards when establishing referral networks, which could be open to abuse, e.g. financial incentives for brokers from providers to promote facilities) [17]. Regulating medical tourist brokers should be a policy priority in both source and destination countries.

## Discussion and directions for future research

**Figure 1:**
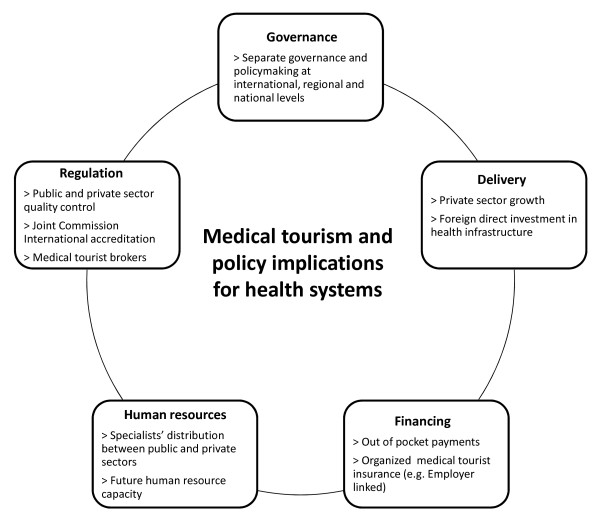
**Conceptual framework for medical tourism and policy implications for health systems**.

Based on the health systems functions of governance, delivery, financing, human resources and regulation [32,33], the conceptual framework (Figure 1) aims to provide a basis for further empirical studies weighing the benefits and disadvantages of medical tourism for health systems, of particular relevance to countries in Southeast Asia.

The framework facilitated the identification of the following variables for empirical analysis:

Governance: the number and content of GATs health sector commitments, the number and size of medical tourist government committees or agencies, availability of medical tourist visa.

Delivery: number of hospitals in public and private sector treating foreign patients, consumption of health services by domestic and foreign population (hospital admissions).

Financing: medical tourist revenues, type of medical tourist payment (service fee or insurance, level of copayment), foreign direct investment in the health sector.

Human resources: doctor and nurse ratios per 1000 population, proportion of specialists in the public and private sectors, number of specialists treating foreign patients.

Regulation: number of JCI accredited hospitals, number of medical tourist visits facilitated by brokers.

At present there is an acute lack of reliable empirical data concerning medical tourist flows. Most urgently, a universal definition of who counts as a medical tourist (e.g. per procedure or per inpatient) should be agreed on, ideally at the international (WHO) or regional level (amongst Ministries of Health, Trade, Tourism and private hospital associations). Variation in definitions and estimates amongst the three study countries alone are significant. Singapore's Tourism Board estimates medical tourist inflows based on tourist exit interviews with a small sample population, whilst the Association of Private Hospitals in Malaysia collects data only from member hospitals and includes all foreign patients, including foreign residents and those who happen to require medical care whilst on vacation [28]. Thailand's Ministry of Commerce collects data on medical tourist inflows from private hospitals, counting foreign patients as the APHM does, except that definitions between hospitals about numbers vary (some count inpatient admissions, others per procedure) [71]. Standardised data collection will enable researchers to make meaningful cross country comparisons, as well as carry out detailed country specific studies to investigate the benefits and disadvantages of medical tourism's impact on health systems.

## Conclusion

The rise of medical tourism in Thailand, Singapore and Malaysia and governments' endorsement of the trend has raised concerns about its potential impact on health systems, namely the exacerbation of existing inequitable resource distribution between the public and private sectors. Nowhere is this more evident than in Southeast Asia, where regulation and corrective policy measures have not kept pace with rapid private sector growth during the past few decades. This paper presents a conceptual framework (Figure 1) that identifies the policy implications of medical tourism for health systems, from a comparative analysis of Thailand, Singapore and Malaysia. This framework can provide a basis for more detailed country specific studies, of particular use for policymakers and industry practitioners in other Southeast Asian countries where governments have expressed an interest in facilitating the development of the industry. Medical tourism can bring economic benefits to countries, including additional resources for investment in healthcare. However, unless properly managed and regulated on the policy side, the financial benefits of medical tourism for health systems may come at the expense of access to and use of health services by local consumers. Governments and industry players would do well to remember that health is wealth for both foreign and local populations.

## Competing interests

The authors declare that they have no competing interests.

## Authors' contributions

NP conducted the research and wrote the first and final versions of the draft. KHP commented on the first and subsequent drafts. NP revised the final manuscript. Both authors read and approved the final manuscript.
